# The Effects of Tetra-Sodium 2-Methyl 1: 4-Naphthohydroquinone Diphosphate on Early Chick and Amphibian Embryos

**DOI:** 10.1038/bjc.1954.74

**Published:** 1954-12

**Authors:** Ruth Bellairs

## Abstract

**Images:**


### THE EFFECTS OF TETRA-SODIUM 2 - METHYL 1:4-NAPHTHO-HYDROQUINONE DIPHOSPHATE ON EARLY CHICK AND AMPHIBIAN EMBRYOS.

RUTH BELLAIRS

*From the Department of Zoology and Comparative Anatomy, St. Bartholomew's Medical College, London, E.C.1.*

Received for publication August 4, 1954.

LOCAL areas of rapidly dividing cells are widely considered to play a part in the development of embryonic organs. The application to developing tissues of substances which interfere with mitosis might therefore be expected to lead to anomalies in the most actively proliferating regions. The action of tetra-sodium 2-methyl-1:4-naphthohydroquinone diphosphate on the early development of the chick has been studied as part of a general investigation into the pathological effects of mitotic inhibitors on growing embryos. A series of experiments has also been carried out on some amphibian embryos. This substance is an analogue of vitamin K manufactured under the trade name of " Synkavit." It has been shown to be an inhibitor of mitosis when applied to cells in tissue culture, and its effects have been described in detail (Mitchell and Simon-Reuss, 1952 ; Hughes and Simon-Reuss, 1953). It has also been used as an aid in the treatment of malignant tumours (Mitchell, 1953).

### MATERIAL AND METHODS.

A total of 109 hens' eggs were used. After an initial period of incubation a small piece of shell and the underlying membrane were removed from the blunt end of each egg ; the stage of the blastoderm was noted and then, in the case of the experimental specimens, 0·5 c.c. of " Synkavit " in solution was injected over the developing embryo. The solution was made up in Pannett and Compton's saline (Pannett and Compton, 1924). Ten control specimens were injected with Pannett and Compton's saline alone, and 6 additional controls were not injected at all. Each egg was resealed with " selotape " and incubated for a further period. The blastoderms were then dissected from the yolk and fixed in either Bouin's or Carnoy's fluid.

Sixty-nine specimens were incubated for 24 hours, and had usually reached the late primitive streak or head process stage before injection. Eight of these were fixed after either 1 or 2 hours' treatment, and the remainder after about 24 hours. Twenty-four eggs were incubated for 48 hours before being injected ; a young embryo had formed by this time in each egg and it possessed a well formed brain, a few pairs of somites and a beating heart ; eight of these specimens were fixed after 24 hours' treatment, whilst the remainder were not examined until 3 days after injection.

Three batches of amphibian eggs were used, two being *Rana temporaria*, the other *Bufo bufo*. Every batch was divided into five equal groups, each consisting of about 50 eggs complete with membranes and jelly. Four groups were placed in identical dishes and immersed in different concentrations of "Synkavit," (1 : 1,000, 1 : 10,000, 1 : 100,000 and 1 : 1,000,000), the solutions being made up with tap water to 200 c.c. in each case. The fifth group in each batch was put into 200 c.c. of tap water alone. The specimens were in early gastrulation at the beginning of the experiment, corresponding to Stages 10 and 11 in the terminology given by Rugh for *Rana pipiens* (Rugh, 1948). The solutions were changed every 24 hours. Six specimens were selected at random from each dish at intervals of about 10–12 hours and fixed in Smith's fluid. Both the chick and amphibian embryos were serially sectioned at 10 $\mu$.

### RESULTS.

The effects of a particular concentration of "Synkavit" on the embryos were not constant, for whilst the development of some individuals appeared to be unaffected, pathological conditions were discovered in others which varied in gravity, some being incompatible with life. With high dosages few chick embryos survived; with low dosages only a small number appeared to be affected. The optimal concentration for causing developmental anomalies was about 1 : 5,000–1 : 10,000 for both chick and amphibian embryos.

It seems likely that individuals varied in their ability to withstand the effects of the drug, but since it was necessary for the "Synkavit" to diffuse through a thin layer of albumen and vitelline membrane in the chick, and through the jelly and membranes in the amphibia, it is also possible that differences in the rate and direction of diffusion may have been largely responsible for the variability of the results.

### *Long term experiments.*

Sixty-one of the chick embryos which had been treated at the primitive streak or head process stage were examined after 24 hours' treatment. Twenty-six had undergone no embryonic development at all, the area pellucida in 16 of these showing various degrees of disintegration. In some cases the area pellucida had completely disappeared and the area opaca had continued to migrate, thus forming a band encircling the yolk. Four embryos had died before the end of the experiment although they appeared to have developed well. Of the 31 embryos which were alive, 9 were apparently normal, whereas the remainder were abnormal in one or more ways. None of the control specimens appeared to have developed abnormally.

---

### EXPLANATION OF PLATE.

FIG. 1.—Transverse section across the hind brain region of a chick embryo after treatment with "Synkavit". The neural folds have failed to close dorsally. The foregut is normal. × 60.

FIG. 2.—Transverse section across the trunk region of the same embryo.
The neural tube is closed, but the heart is developing as two separate vesicles. × 60.

FIG. 3.—Enlargement of a part of the neural plate in Fig. 1. Resting cells are not found along the free edge at the base of the neural groove. Numerous resting cells can be seen further dorsally along the free edge and here the neural plate becomes convex. *nor.* = normal pattern. × 270.

FIG. 4.—*Rana temporaria* embryo treated with "Synkavit" showing open neural grooves. × 14.

FIG. 5.—*Rana temporaria* control embryo, of the same stage as Fig. 4. showing closed neural grooves. × 14.

FIG. 6.—Transverse section of hyperplastic patch in embryo of *R. temporaria*. The epithelium has become multi-layered. *n.* = nuclei; *i.c.* = intra-epidermal cyst. × 85.

Bellairs.

(a) *Neural tissue.*—The most frequently obtained anomaly was a failure of the neural folds to close dorsally. Out of 31 well formed chick embryos 17 showed failure of the neural folds to close (Fig. 1). In 5 of these this defect extended the whole length of the embryo; in the remainder it was confined to the hind brain region, the neural folds being closed both anteriorly and posteriorly to this level (Fig. 2). In the controls and in those experimental specimens where the neural tube was completely or almost closed the histological pattern was normal (Altmann, 1885; Rabl, 1918; Sauer, 1935, 1936); it consisted of a thickened epithelium of tall columnar cells with nuclei near the base. The numerous dividing cells had rounded off and moved towards the free edge. Resting nuclei were seldom found along this border. In embryos in which the neural plate had remained flattened, however, both resting and dividing cells were found along the free edge. In specimens where the neural tube had closed except in the hind brain region, the histological structure differed in the two parts; the normal and pathological pattern could even be seen in the same section (Fig. 3).

Failure of the neural folds to close was also obtained with the amphibian embryos (Fig. 4). In these specimens the stage of development corresponded with that of the controls after the same length of time even though the degree of closure of the neural tube lagged behind (Fig. 5). In one series after 28 hours' treatment with " Synkavit " the neural folds were widely open in the hind brain region in every one of 38 specimens, whereas they were all practically closed in the control embryos. After a total of 65 hours the neural folds had fused in about half the treated embryos. Hatching occurred at approximately this time in both the treated and control series. The larvae with open neural plates were capable of swimming movements though they tended to be less energetic than the others and did not grow so quickly; no further healing occurred and indeed the condition tended to deteriorate, a white mass exuding from the malformed region. Inspection of serially sectioned material showed that this was due to disintegration of the floor of the neural groove. The neural crest and the spinal ganglia appeared to be normal. The epidermis, however, tended to be raised up in patches alongside the open neural plate and on the flanks. Inspection of serial sections revealed a hyperplasia of the epidermis in these regions. The individual cells did not appear to be enlarged or œdamatous, but they were arranged in strata instead of in the normal single-layered manner. Large intra-epidermal cysts which presumably contained fluid, were found among them (Fig. 6). A similar phenomenon occurred infrequently in the chick embryos (5 cases).

(b) *Size.*—Some of the more severely affected chick embryos were grossly stunted. Where good differentiation occurred however, even though abnormalities were found, the size usually appeared to be not much less than that of the control specimens, e.g. 17 chick embryos which had been treated at the primitive streak or head process stage and fixed after a further 24 hours' incubation, ranged in length from 910$\mu$–4000$\mu$ (mean 2, 670$\mu$), whereas 6 control specimens fixed at the same time were between 2070$\mu$ and 4650$\mu$ in length (mean 2920$\mu$). Similarly among the amphibian embryos only those specimens in which the failure of the neural plate to close was most pronounced tended to be shorter at the time of hatching.

(c) *Heart, gut and blood vessels.*—In 5 of the chick embryos the heart failed to develop, in 10 it differentiated as two separate vesicles (Fig. 2), whilst in the remaining 16 it was present as a single ventral organ beneath the foregut. In the region where the two vesicles remained apart the foregut floor was absent. No correlation was found between the failure of the neural tube to close dorsally in the hind brain region and failure of the heart vesicles and the floor to fuse ventrally, for 6 of the embryos which had open neural plates had a well formed, single heart, whereas 4, which had a normal dorsal aspect, possessed either two heart vesicles or no heart at all. In 5 cases the normal single-layered cubical epithelium of the presumptive foregut floor (Bellairs, 1953) was replaced in certain regions by a multi-layered arrangement. In 2 others the presumptive foregut floor though single-layered was abnormally thin.

The development of the heart in the amphibian embryos did not appear to be affected. In both the chicks and the amphibians, the paired dorsal aortae apparently developed normally. Somites and notochord were always present, and there was no apparent deficiency of the head mesenchyme. The differentiation of the area vasculosa in the chick was disturbed, however, in some of the older specimens which were treated after about 48 hours of incubation (at about Stage 13 of Hamburger and Hamilton, 1951). Eight specimens were examined 24 hours after treatment, and in 5 of these the region was pale with few blood vessels, especially at the posterior end. Sixteen further embryos were inspected after 3 days, and in 9 of these vascularisation in the area vasculosa was reduced in extent or absent. Six untreated control specimens were fixed after 3 days, and in only 1 of these did the area vasculosa appear to be retarded.

The expansion of the extra-embryonic tissues of the blastoderm over the surface of the yolk appeared to be unaffected by the " Synkavit "; measurements taken at the time of treatment (19 experimental specimens and 6 control ones) and after a further 24 hours showed that in both groups the region had increased between 2 and 3 times in diameter. In no case was an open meshwork obtained of the type formed after treatment with folic acid antagonists (Bellairs, 1954).

(d) *Individual cells.*—In most of the specimens fixed 24 hours after application of " Synkavit ", little indication of its pathological effects on the cells could be discovered. In 11 embryos some of the chromosomes were seen clumped together in metaphase ; occasionally chromosomes were found to be scattered through the cytoplasm. These anomalies have, however, also been seen in untreated embryos, though rarely. In all cases all phases of the mitotic cycle were present.

The proportions in which the various phases were found were similar to those of comparable normal embryos. Three typical " Synkavit "-affected specimens were selected, each having an open neural plate, short foregut and two separate hearts ; mitotic counts were made in the endoderm of every third section. In each case dividing cells were more frequently seen in metaphase than in prophase, the percentages being in each specimen respectively : 1. P 31 per cent ; M 49 per cent. ; 2. P 25 per cent : M 64 per cent ; 3. P 32 per cent : M 48 per cent. The distribution of mitotic phases in 3 untreated control embryos was not substantially different from these values. The mitotic rate throughout the endoderm as a whole was rather higher in the treated (range 2·15–2·32 per cent) than in the control embryos (range 0·9–1·65 per cent).

*Short term experiments.*

Short term experiments were carried out to investigate some of the cellular changes which may be attributed to the immediate and direct action of "Synkavit".

The 8 specimens in this group were all injected after 24 hours' preliminary incubation; 4 were fixed 1 hour after application of the drug, and 4 after 2 hours. Sections were examined under oil immersion. Most of the cells, both dividing and resting, appeared to be normal, and all phases of mitosis were present in each case; some evidence of interference with mitosis, however, was seen in 6 of the specimens. Cells were occasionally found in which the nuclear membrane had disappeared and the chromosomes were scattered throughout the cytoplasm. Some large swollen cells were also discovered which possessed big, pale nuclei and, infrequently, vacuolated cytoplasm. Clumping of the chromosomes in metaphase was rarely seen.

### DISCUSSION.

When studying the effects of the mitotic inhibitors on developing embryos it is important to determine firstly that some interference with cell division has occurred, and secondly whether the anomalies obtained could be explained in terms other than of mitotic inhibition. The present investigation shows that there is evidence of mitotic inhibition in the chick after treatment with " Synkavit " ; effects on dividing cells were found after about 2 hours' treatment which were similar to those reported by Mitchell and Simon-Reuss (1952) for cells in tissue culture.

After 24 hours' treatment with " Synkavit," however, few abnormalities could be seen, these being confined mainly to clumping of the chromosomes in metaphase; a similar result was obtained by Mitchell and Simon-Reuss (1952). It seems likely that the cells had recovered from the effects of the drug. There is some evidence to show that a reduction in the inhibitory effects of the drug on mitosis may have also occurred ; Mitchell and Simon-Reuss (1952) have shown that if " Synkavit " is kept at 39° C. before being applied to cells in tissue culture, there is a progressive reduction of its anti-mitotic activity with increasing length of time. The greatest amount of mitotic inhibition in the present experiments would therefore be expected to take place soon after application of the drug.

The fact that the proportions of the various mitotic phases did not differ in specimens treated for 24 hours with " Synkavit " from those of comparable controls was also found by Mitchell and Simon-Reuss (1952) and by Hughes and Simon-Reuss (1953). Hughes and Simon-Reuss concluded that "no single phase of mitosis is especially sensitive to the influence of " Synkavit "."

In addition to the inhibitory effects of " Synkavit" on mitosis, however, the occasional vacuolation of the cytoplasm in the chick cells suggests that some of the pathological anomalies may have owed their origin to some other cause. Cytoplasmic vacuolation was also reported by Mitchell and Simon-Reuss (1952) and by Hughes and Simon-Reuss (1953).

The reduced size of the more grossly affected embryos might at first appear to be a direct result of the interference with cell division. Since specimens which were only slightly malformed tended to be of a size comparable with the controls it could be postulated these quickly overcame the inhibitory effects, or that the drug rapidly became inactivated, and that the embryos were thus soon able to continue their growth and differentiation in the normal way. It is, however, not impossible that the smallness of the severely disturbed specimens was accentuated by their reduced ability to carry out metabolic processes as compared with the less disturbed embryos. There is some evidence that this may be so in the amphibian embryos, for after hatching they failed to increase in size as rapidly as the normal larvae. It is strange, moreover, that if the small size of the grossly disturbed embryos is due entirely to a reduction in the rate of cell division, this phenomenon has not apparently affected the extra-embryonic tissues in the chick. Bellairs (1954) has shown that after heavy doses of folic acid antagonists the area opaca, whilst continuing to expand over the surface of the yolk, tears itself into holes; this was deduced to be the result of the continued expansion of that region in the face of an inadequacy in the cell population.

The inhibition of blood-island formation in the area vasculosa of the chick embryos may have been due to a partial inhibition of cell division in the splanchnic mesoderm. It is strange that this effect persisted up to the third day, however, for considerable recovery of the affected cells and a reduction in the inhibitory powers of the drug would be expected to have taken place during this time. It seems possible, therefore, that some effect in addition to mitotic inhibition may have influenced the development of the area vasculosa.

The failure of the neural folds to close off dorsally is a phenomenon which can be produced with some regularity in embryos by the application of diverse techniques, e.g. by ultra-violet irradiations (Davis, 1944), by lithium (Jenkinson, 1906; Ogi, 1954), by trypan blue (Gillman, Gilbert and Gillman, 1948), or by concentrated salts and glycerine (Hobson, 1941). It is unlikely that the mechanism involved in each case is an interference with mitosis. It is difficult to ascertain, therefore, whether reduced cell division is the only cause for the failure to close of the neural plates in the present experiments. Ancel (1945, 1946) is of the opinion that when teratogenous substances which produce *spina bifida* are applied to chick embryos of about 26 hours' incubation they act primarily on the underlying mesoderm, which consequently fails to bring about normal neural induction. In the present experiments, however, there was no reduction in the amount of mesoderm present, nor did it appear to be malformed in this region.

The failure of dorsal closure is unlikely to be the result of a failure of the neighbouring ectoderm to expand sufficiently to permit the process, since the latter often possess hyperplastic patches, at least in the amphibians.

Evidence from other sources as to the factors responsible for neural tube closure is contradictory, though many authors believe that changes in cell shape rather than in cellular proliferation are important. Mitotic nuclei are found only along the free edge of the neural plate because the cells round up and move into that position in order to divide (Altmann, 1885; Rabl, 1918; Sauer, 1935, 1936). After division the two daughter cells elongate and their nuclei migrate back to the basal layer. Boerema (1929) using amphibian embryos has described changes in cell shape, the ectodermal cells becoming taller and more wedge-shaped as neural tube closure proceeds. It is of interest therefore that in the present experiments the histological arrangement of the chick neural tissue differs between neural tubes and open neural plates even in the same embryo. It is suggested that where the neural plate has failed to close there was an interruption of the process of cell division which had repercussions on the shape of individual cells. It is known that cells which have been interrupted during mitosis may sometimes reform a resting nucleus (Hughes, 1950). It seems possible that, such an interruption having occurred in the neural plate, the reformed nuclei do not return to their original position in the basal layer and their cytoplasm fails to reassume a columnar structure. Such a process would lead to an increase in the width of the free as opposed to the basal edge of the neural plate, and could therefore result in a failure to close to a tube. If this is so, however, it poses the difficult question as to why the reconstructing nuclei fail to return to the basal layer.

A similar explanation was put forward to account for the frequent failure to form the foregut floor in chick embryos after treatment with folic acid antagonists (Bellairs, 1954). In the present experiments, however, such abnormalities in the histological pattern of the foregut floor were found in only 5 specimens, and in each case the extent of the region with arrested or reconstructed mitoses was so restricted that it cannot be regarded as a prime cause of the failure to close. The reason for the production of this anomaly in the chick embryos, after treatment with " Synkavit," together with the frequent failure of the two heart vesicles to unite, is obscure, and the possibility cannot be excluded that some action of the drug other than mitotic inhibition may have contributed to it.

The pathological effects produced in chick embryos by the application of a number of different mitotic inhibitors are not constant, e.g. somites frequently failed to develop when chick embryos were treated with aminopterin (Bellairs, 1954), but not when colchicine (Bellairs, 1954) or " Synkavit " were applied. Strophosomy may be produced when blastoderms are treated after 48 hours' initial incubation with colchicine (Lallemand, 1939 ; Bellairs, 1954, unpublished), but not with " Synkavit ". This lack of unity may in part be due to a failure to employ comparable concentrations of the drugs, and to the fact that whereas the dividing cells are able to recover from the effects of certain inhibitors (e.g. folic acid antagonists and " Synkavit "), their further development is completely impeded with others (e.g. colchicine). When doses sufficiently high to inhibit all axis formation are used, however, the effects are still not constant, aminopterin leading to the development of an open meshwork in the blastoderm, especially in the area opaca (Bellairs, 1954), " Synkavit " frequently causing a dissolution of the area pellucida; excessive doses of colchicine usually produced small dense blastoderms, irregular in shape and with no hole in them; frequently they were only partially fixed to the vitelline membrane and the free edge had become folded over, ectoderm to ectoderm (Bellairs, 1954, unpublished).

The lack of conformity of results obtained with substances often considered to be " mitotic inhibitors," could only be interpreted if more were understood about the biochemical processes which are disturbed by these agents. Nothing is known about the chemical mode of action of either colchicine or " Synkavit ". It is not possible therefore to analyse the observations recorded above, beyond the statement that although some of the effects are probably the result of mitotic inhibition, others may have been caused in other ways. In this connection it is of interest that " Synkavit " may have at least two quite different effects on cells; on the one hand it is required by liver cells in the synthesis of prothrombin, a protein with a highly specific function, and on the other, it sensitises some malignant cells to the effects of ionising irradiation (Mitchell, 1953).

### SUMMARY.

1. Chick and amphibian embryos have been treated during gastrulation with a mitotic inhibitor, tetra-sodium 2-methyl 1:4-naphthohydroquinone diphosphate.

2. The effects of this compound on the mitotic cells are described.

3. The most frequent morphological anomaly was that the neural folds failed to close in the region of the presumptive hind brain. It is suggested that interference with cell division led to a change in the histological structure of the neural plate, the width of the free distal surface failing to become less than that of the basal part.

4. Other anomalies in the chick were failure of the two sides of the foregut floor and the two heart vesicles to unite ventrally, reduction in blood island formation and in the size of the embryos. It is concluded that these anomalies were not solely due to an interference with cell division.

This work was begun in the Anatomy Department of the University of Cambridge at the suggestion of Dr. A. F. W. Hughes. I am most grateful to Mrs. I. Simon-Reuss, not only for gifts of " Synkavit " but also for reading the manuscript. Mr. M. Abercrombie also kindly read the manuscript. I am indebted to Miss O. Wilkinson and Mr. J. F. Fozzard, F.R.P.S., for assistance with the photographs.

### BIBLIOGRAPHY.

Altmann, R.—(1885) *Arch. Anat. Physiol., Lpz.*, Anatomie, 344.
Ancel, P.—(1945) *C. R. Soc. Biol., Paris*, **139**, 984.—(1946) *Ibid.*, **140**, 320.
Bellairs, Ruth.—(1953) *J. Embryol. exp. Morph.*, **1**, 115.—(1954) 'Ciba Symposium on Chemistry and Biology of Pteridines.' (In the press).
Boerema, I.—(1929) *Arch. EntwMech. Org.*, **115**, 601.
Davis, J. O.—(1944) *Biol. Bull., Wood's Hole*, **87**, 73.
Gillman, J., Gilbert, C. and Gillman, T.—(1948) *S. Afr. J. med. Sci.*, **13**, 47.
Hamburger, V. and Hamilton, H. L.—(1951) *J. Morph.*, **88**, 49.
Hobson, L. B.—(1941) *J. exp. Zool.*, **88**, 107.
Hughes, A. F. W.—(1950) *Quart. J. micr. Sci.*, **91**, 251.
Hughes, A. and Simon-Reuss, I.—(1953) *Brit. J. Cancer*, **7**, 142.
Jenkinson, J. W.—(1906) *Arch EntwMech. Org.*, **21**, 367.
Lallemand, S.—(1939) *Arch. Anat., Strasbourg*, **28**, 215.
Mitchell, J. S.—(1953) *Brit. J. Cancer*, **7**, 313.
*Idem*, and Simon-Reuss, I.—(1952) *Brit. J. Cancer*, **6**, 317.
Ogi, K.—(1954) *Sci. Rep. Tôhoku. Univ.*, **20**, 163.
Pannett, C. A. and Compton, A.—(1924) *Lancet*, i, 381.
Rabl, C.—(1918) *Arch. mikr. Anat.*, **90**, 261.
Rugh, R.—(1948) '*Experimental Embryology*, Minneapolis, (Burgess).
Sauer, F. C.—(1935) *J. comp. Neurol.*, **62**, 377.—(1936) *Ibid.*, **63**, 13.


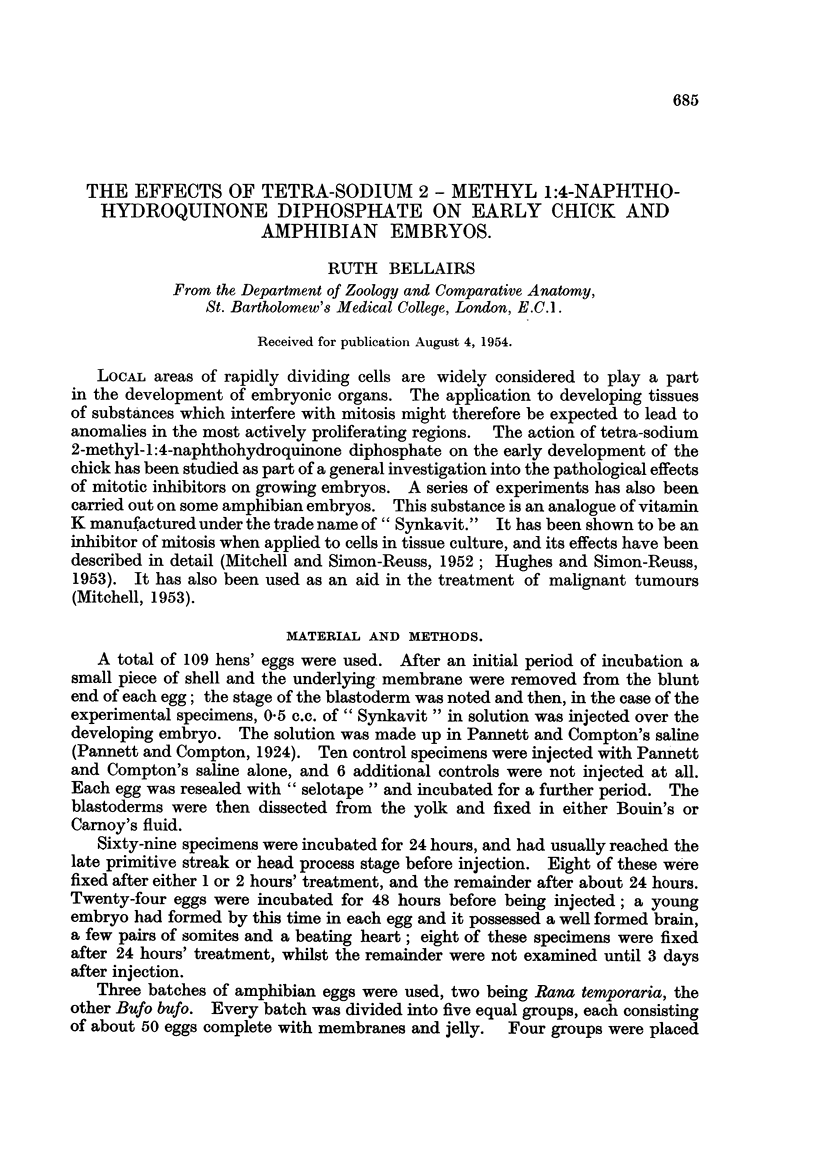

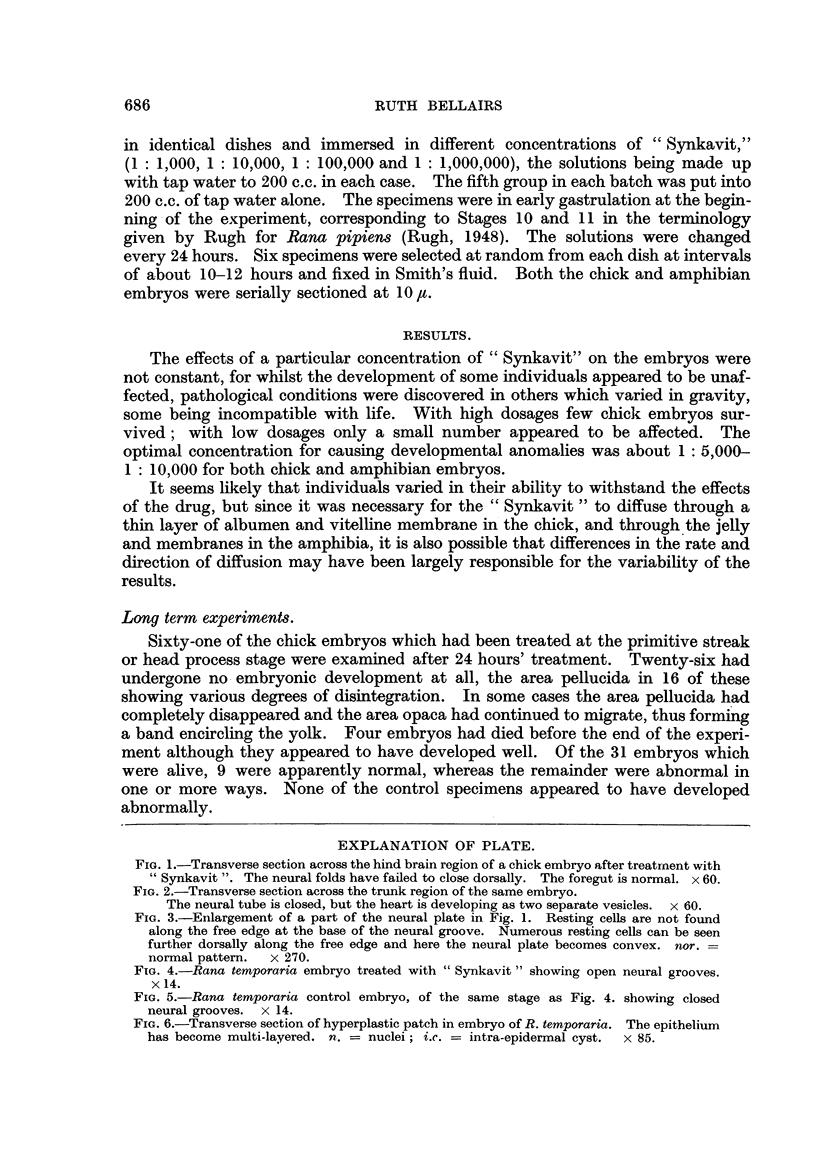

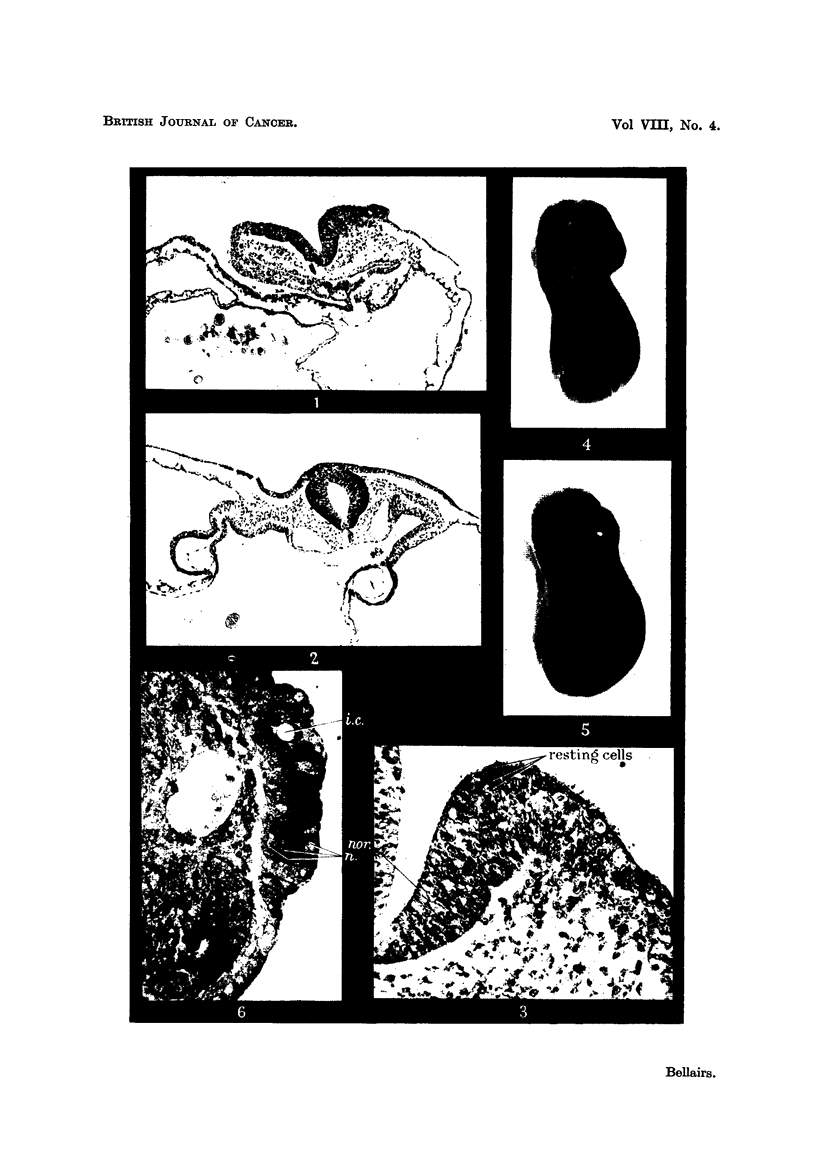

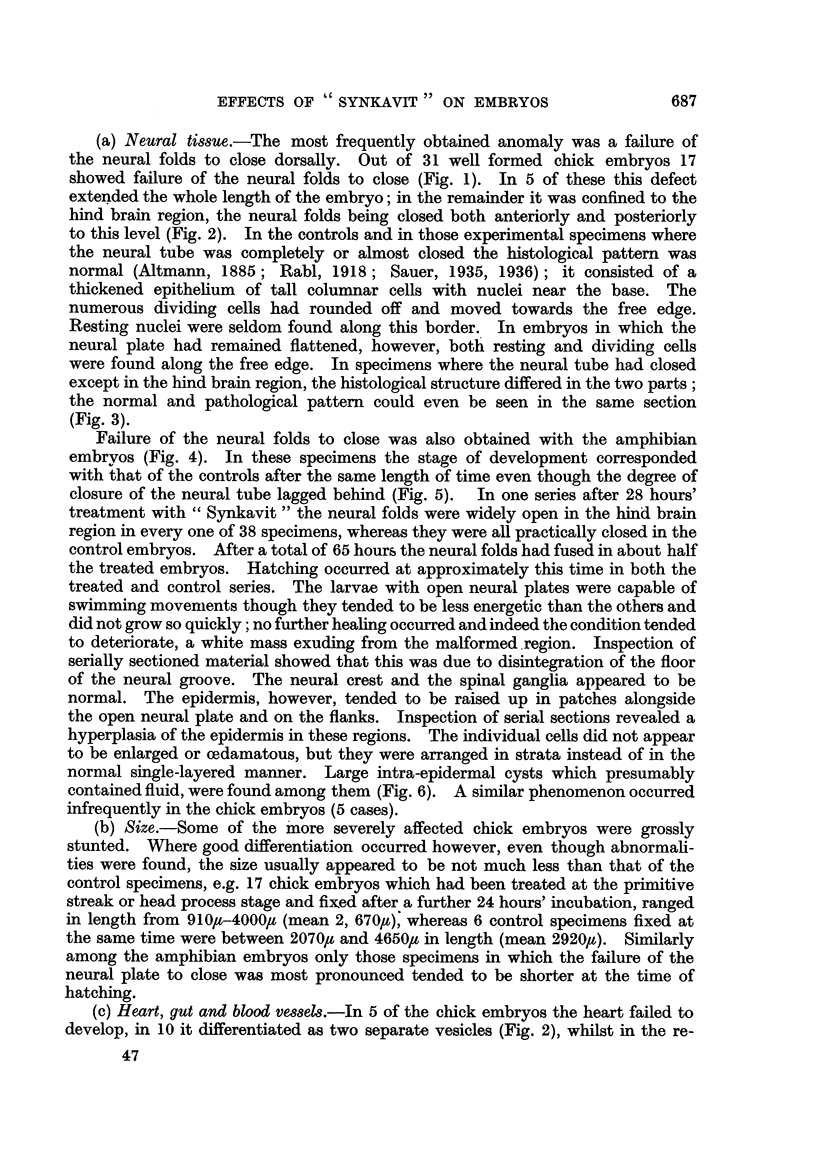

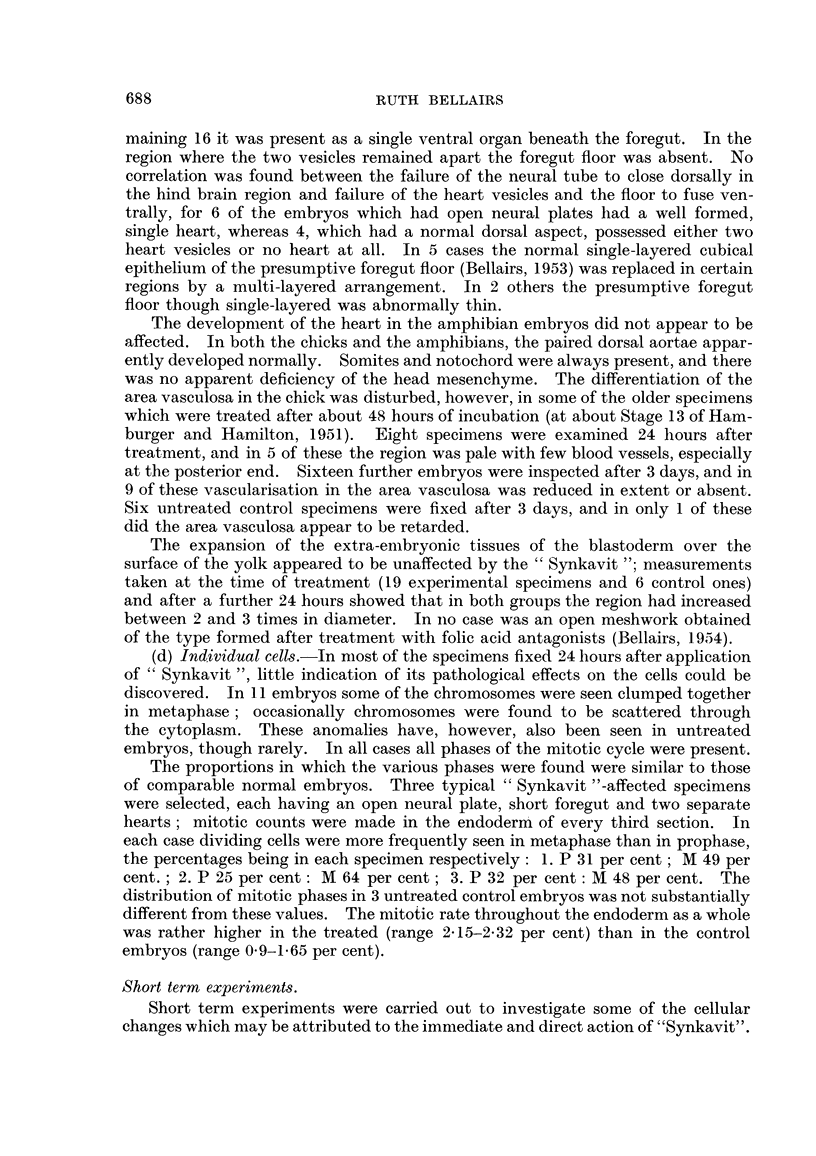

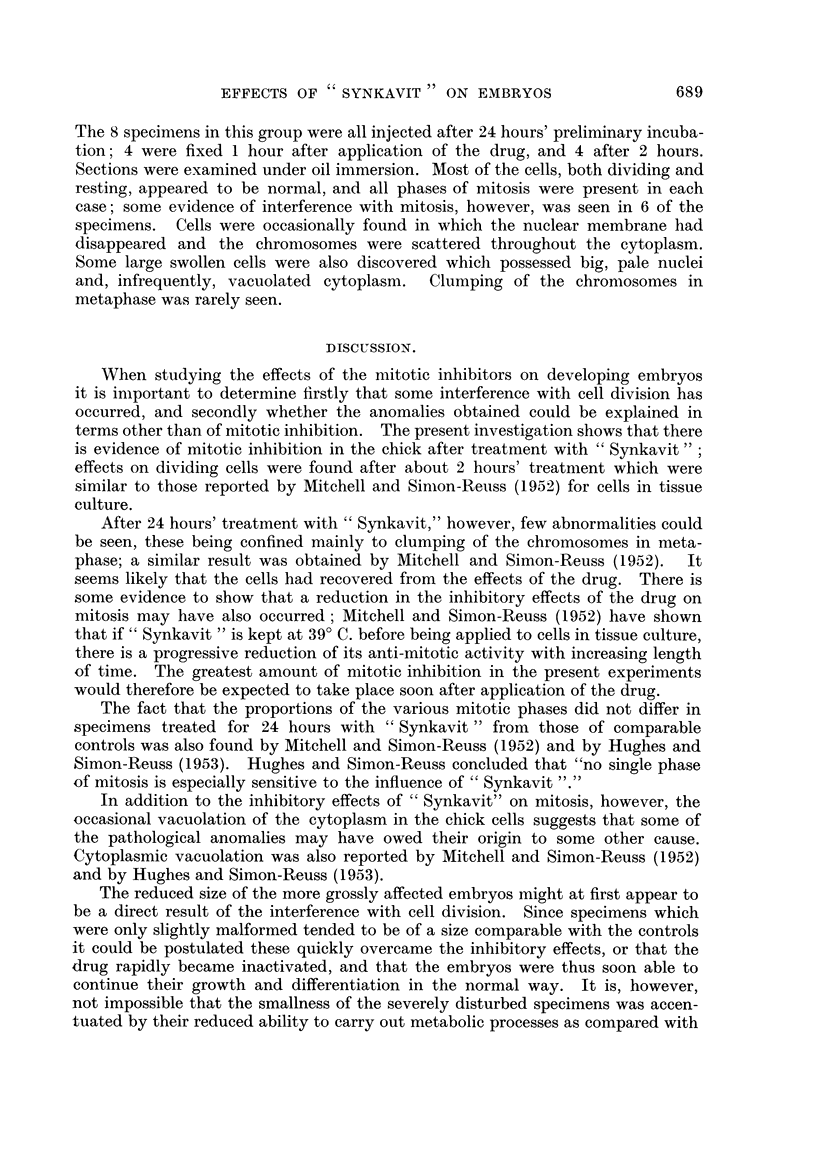

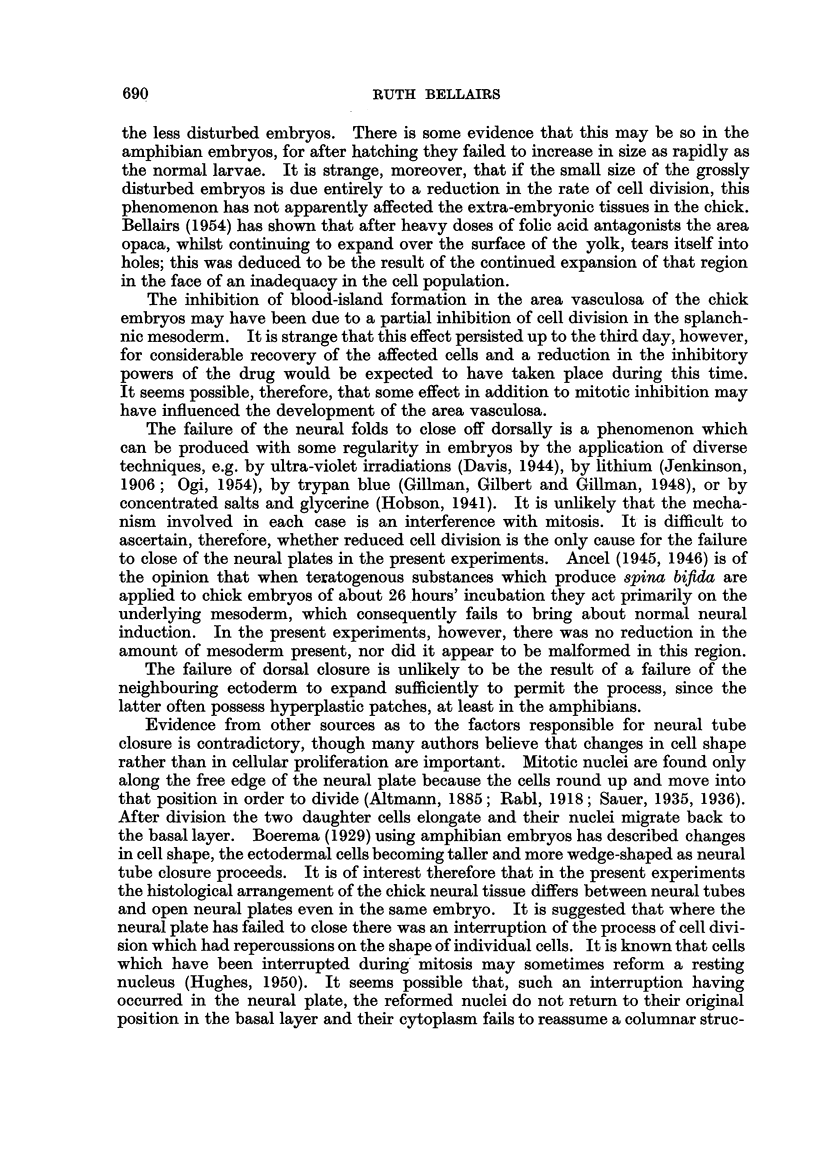

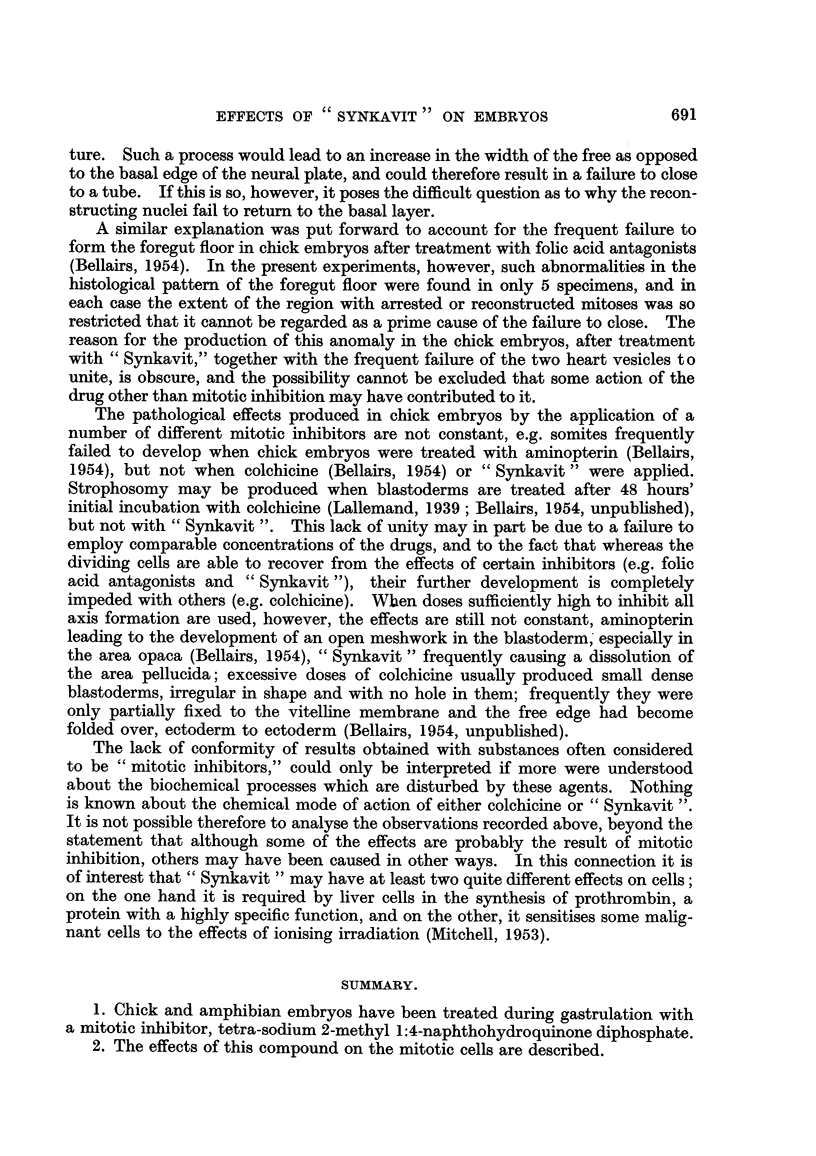

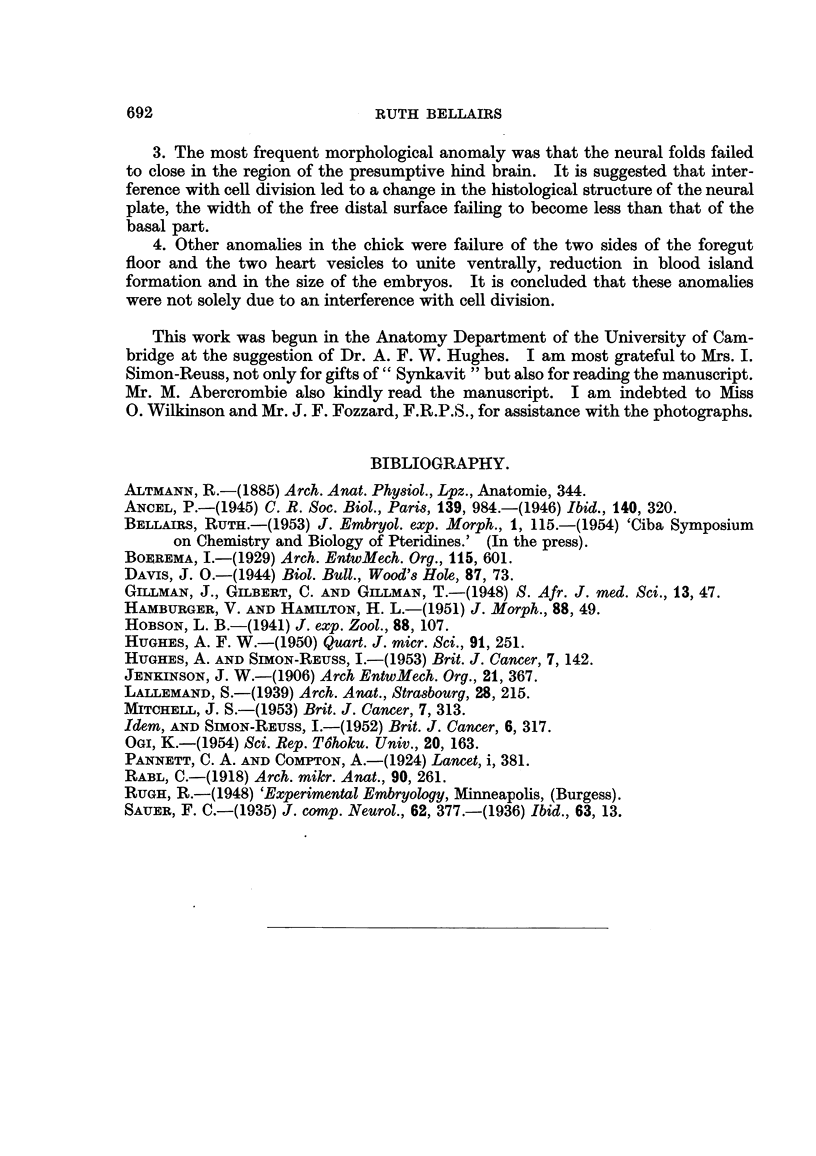


## References

[OCR_00486] HUGHES A., SIMON-REUSS I. (1953). The immediate effect of some phosphorylated naphthohydroquinones on dividing cells; a study on living cultures of chick osteoblasts.. Br J Cancer.

